# Mid-term clinical outcomes and isokinetic performance after primary total knee arthroplasty within a modified ERAS protocol: a single-blinded randomized controlled trial

**DOI:** 10.1186/s12891-026-10535-3

**Published:** 2026-10-22

**Authors:** Julia Schiegl, Felix Greimel, Florian Zeman, Joachim Grifka, Tobias Renkawitz, Franziska Leiss

**Affiliations:** 1 Department of Orthopaedics, Regensburg University, Asklepios Klinikum Bad Abbach, Kaiser-Karl V. Allee 3, 93077 Bad Abbach, Germany; 2 Orthopaedics Regensburg MVZ- Specialist center for orthopaedics and trauma surgery -Im Gewerbepark C10, 93059 Regensburg, Germany; 3 Center for Clinical Studies, University Medical Center Regensburg, Franz-Josef- Strauss-Allee 11, 93053 Regensburg, Germany; 4 Regensburg University of Applied Science, Seybothstraße 2, 93053 Regensburg, Germany

**Keywords:** Biodex^®^, Early mobilization, Enhanced recovery after surgery (ERAS), Isokinetic dynamometer, Isokinetic muscle strength, Mid-term outcome, Total knee arthroplasty (TKA)

## Abstract

**Background:**

Persistent muscular deficits are a major contributor to functional limitations following total knee arthroplasty (TKA). While Enhanced Recovery After Surgery (ERAS) protocols are increasingly adopted to improve postoperative outcomes, their influence on mid-term objective muscle strength recovery remains insufficiently defined. This study aimed first, to determine whether a modified ERAS protocol enhances mid-term recovery of knee muscle strength after primary TKA and second, compare associated clinical outcomes using validated functional and patient-reported measures.

**Methods:**

The study received ethical approval from the Ethics Committee of the University of Regensburg, Germany (Reference No. 19-1470-101 on 31 July 2019) and was conducted in accordance with the principles of the Declaration of Helsinki. In this prospective, single-blinded randomized controlled trial, 58 patients undergoing primary TKA were assigned to either a modified ERAS protocol (*n* = 31) or conventional perioperative care (*n* = 27). Assessments were conducted at 5 days, 4 weeks, 3 months, 6 months, and 12 months postoperatively. Isokinetic muscle strength - including peak torque (Nm), total work (J), and power (W) - was measured using the Biodex^®^ System 3 Dynamometer. Clinical outcomes were evaluated using the Numeric Rating Scale (NRS) for pain, Patient-Reported Outcome Measures (PROMs), and the Knee injury and Osteoarthritis Outcome Score (KOOS).

**Results:**

At 5 days postoperatively, the ERAS group demonstrated significantly higher isokinetic muscle strength and improved active knee flexion compared to the conventional group (*p* < 0.05). These early differences decreased over time, with no significant differences observed between groups at 3, 6, or 12 months. Both groups demonstrated excellent mid-term outcomes, with similarly high patient satisfaction and comparable PROMs and KOOS scores at the one-year follow-up.

**Conclusion:**

The modified ERAS protocol led to significantly improved early postoperative recovery of knee muscle strength without adversely affecting short- or mid-term outcomes. Although isokinetic and clinical measures aligned across groups over time, the ERAS approach appears to be a safe and effective strategy for enhancing early functional recovery and overall patient experience following primary TKA.

**Supplementary Information:**

The online version contains supplementary material available at https://doi.org/10.1186/s12891-026-10535-3.

## Background

Enhanced Recovery After Surgery (ERAS) protocols have gained increasing prominence in orthopaedic surgery, particularly for total knee arthroplasty (TKA). Originally developed for colorectal procedures, ERAS aims to optimize perioperative care and accelerate functional recovery through a multimodal approach, that includes early mobilization, optimized pain control, minimally invasive techniques, and standardized anaesthesia and fluid management strategies [1–3]. In the context of TKA, ERAS has been associated with reduced morbidity, lower complication rates, shorter hospital stays, and improved early function [3–8]. These benefits are becoming increasingly relevant as TKA procedure volumes continue to rise globally [9, 10]. 

Despite its overall success, up to 10% of TKA patients report postoperative dissatisfaction [11]. One of the main contributors to this dissatisfaction is persistent muscular weakness, especially the quadriceps and hamstrings, which impairs mobility and delays rehabilitation [12, 13]. ERAS protocols aim to counteract these effects by promoting early ambulation and minimizing the catabolic stress response to surgery. Several studies have demonstrated that early mobilization reduces thromboembolic risk and accelerates functional recovery [5, 8, 14–16]. Isokinetic testing using devices like the Biodex^®^ System is considered the gold standard for objective assessment of knee muscle function [17, 18]. A recent randomized controlled trial in total hip arthroplasty (THA) found that patients managed with a modified ERAS protocol demonstrated significantly higher peak torque, total work, and power at 3 months postoperatively, with sustained improvements in total work at 1 year - without compromising clinical outcomes [19]. However, similar mid-term data for TKA patients are lacking, particularly regarding the relationship between objective strength outcomes and patient-reported measures such as KOOS [20, 21]. Patient-reported outcome measures (PROMs), the Knee Injury and Osteoarthritis Outcome Score (KOOS), numeric rating scale (NRS), and patient satisfaction scores are equally essential for capturing both functional outcome and patient experience [16]. Recent studies have highlighted minimal clinically important differences (MCID) and potential ceiling effects in KOOS domains, underscoring the importance of comprehensive follow-up to meaningfully interpret PROMs [22, 23]. Furthermore, combined low KOOS scores and poor mental health have been shown to predict dissatisfaction after TKA [24].

Although early postoperative isokinetic outcomes within the present randomized cohort have previously been reported [8], the persistence of these effects beyond the immediate postoperative period remains unclear. In particular, longitudinal data integrating serial isokinetic muscle performance with validated patient-reported outcomes over a follow-up period of up to one year are lacking. The present study therefore addresses whether the early functional advantages observed with the modified ERAS pathway translate into sustained differences in objective muscle performance and patient-reported outcomes during mid-term recovery after TKA.

### Aim of the study

The primary objective of this prospective, single-blinded randomized controlled trial was to determine whether the early postoperative differences observed between the modified ERAS and conventional care groups persist during mid-term recovery. Specifically, isokinetic muscle performance, including peak torque, total work, and power, was compared between groups over a follow-up period of one year after primary TKA.

The secondary objective was to evaluate whether differences in objective muscle performance were accompanied by corresponding differences in patient-reported and clinical outcomes, including pain perception (NRS), self-perceived health, expectation fulfilment, and KOOS subscale scores. By integrating serial objective performance testing and PROMs across multiple postoperative time points, the present study extends the previously reported early postoperative analysis and provides a comprehensive assessment of recovery within modified ERAS versus conventional TKA pathways.

## Methods

### Study design and patient allocation

This prospective, randomized, single-blinded controlled trial included 58 patients who underwent primary cemented TKA between May 2020 and March 2021 at an orthopaedic university clinic specializing in high-volume arthroplasty. Participants were randomized preoperatively using sealed envelopes by an independent statistician: 31 patients were allocated to the ERAS group and 27 to the conventional group. The study was single-blinded with respect to the patients, who were not informed of their treatment allocation. To minimize interaction between the treatment groups and reduce the possibility of identifying group allocation through comparison of perioperative management, patients assigned to the ERAS and conventional-care pathways were accommodated on separate wards located on different floors.

### Inclusion and exclusion criteria

Inclusion criteria comprised adults undergoing primary cemented, navigated TKA with the P.F.C.™ Sigma system (DePuy Synthes, Johnson & Johnson, Raynham, MA, USA) due to primary or secondary osteoarthritis. All procedures were carried out within the concept of mechanical alignment.

Exclusion criteria included: age < 18 or > 90 years; history of deep vein thrombosis; ongoing therapeutic anticoagulation; limited preoperative mobility (walking distance < 100 m with aids); prior surgery or tumor at the operative site; BMI > 50; recent open surgery (excluding arthroscopy); simultaneous enrolment in another study; or refusal to participate.

### Ethics approval

The study protocol received approval from the responsible Ethics Committee of the University of Regensburg, Germany (Reference No. 19-1470-101), on July 31, 2019, before enrolment of the first participant. No modifications to the study protocol, treatment allocation, assessment schedule, or planned outcome measurements were made after initiation of the trial. The study was registered in the German Clinical Trials Register (DRKS; DRKS00041069) on July 23, 2026, prior to submission of the manuscript. The study was conducted in accordance with the principles of the Declaration of Helsinki. All participants received detailed information about the study and provided written informed consent before participation, including consent for the publication of anonymized data. This manuscript contains no identifying personal information.

The primary study objective was to evaluate differences in postoperative recovery of objective isokinetic knee muscle function between patients treated within the modified ERAS pathway and those receiving conventional perioperative care. Isokinetic outcomes comprised peak torque, total work, and power during knee flexion and extension, assessed using the Biodex^®^ System 3 Dynamometer.

Secondary outcomes comprised clinical and patient-reported measures of postoperative recovery, including pain intensity (NRS), active knee range of motion, KOOS subscale scores, patient-reported success of surgery, willingness to undergo the procedure again, fulfilment of preoperative expectations, perceived change compared with the preoperative condition, and perceived improvement in quality of life.

### Surgical and perioperative protocols

**Fig. 1:**
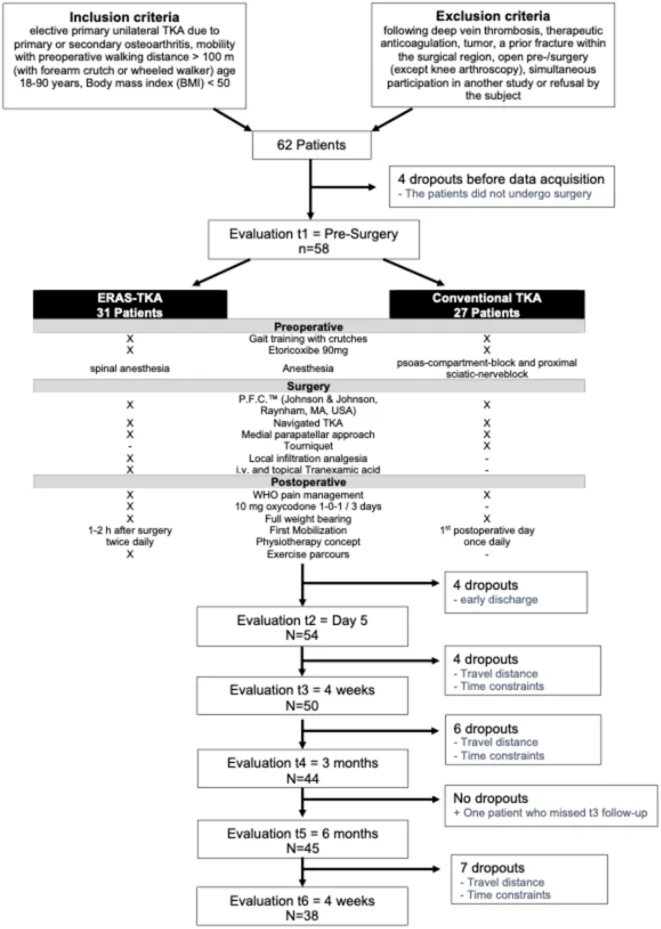
CONSORT participant-flow diagram illustrating the study design, patient allocation, and follow-up. Assessments were conducted preoperatively (T1) and postoperatively at 5 days (T2), 4 weeks (T3), 3 months (T4), 6 months (T5), and 1 year (T6). Loss to follow-up was primarily due to patients declining further assessments because of travel distance and time constraints. No patients were lost to follow-up due to revision surgery, postoperative complications, or death

All surgeries were performed at an academic, high-volume arthroplasty center. Figure 1 summarizes key differences between the ERAS and conventional care pathways.

Both groups underwent TKA using a medial parapatellar approach with cemented components from the same manufacturer. Patellar resurfacing was not performed in any patient in either treatment group. Immediate full weight-bearing was permitted postoperatively.

Perioperative antibiotic prophylaxis was identical in both treatment groups. All patients received antibiotic prophylaxis according to the institutional standard, with cefazolin (2 g) administered intravenously approximately 30 min before skin incision. In patients with a known cephalosporin allergy, clindamycin (600 mg) was administered intravenously as an alternative. A second dose of antibiotics could be administered in cases of prolonged operative duration at the discretion of the operating surgeon. Routine postoperative antibiotic prophylaxis was not administered. This approach is consistent with the short-duration perioperative antibiotic prophylaxis recommended for elective primary hip and knee arthroplasty by Gorišek Miksić et al. [25].

### Component alignment

All procedures were performed according to a mechanical alignment philosophy using computer-assisted navigation. Tibial component rotation was determined intraoperatively using standardized anatomical landmarks. The tibial component was positioned in external rotation relative to Akagi’s line and oriented toward the medial third of the tibial tuberosity. Following insertion of the trial components, three full flexion–extension cycles were performed, and the final rotational position of the tibial component was determined based on this assessment.

### ERAS group

Patients received preoperative interdisciplinary education, gait training, and preemptive analgesia with etoricoxib (90 mg). Spinal anaesthesia was administered using hyperbaric prilocaine (80 mg), and sufentanil (10 µg), along with dexamethasone (8 mg) intravenously. Surgery was performed without a tourniquet. Local infiltration analgesia included ropivacaine (200 mg) with epinephrine, and tranexamic acid (1 g IV + 2 g topical). Wounds were closed without drains and covered with transparent film dressings. Patients were mobilized within 1–2 h post-surgery, targeting an initial walking distance of 50 m. Postoperative therapy consisted of twice-daily physiotherapy with focusing on gait correction, coordination, and strengthening.

### Conventional group

Patients received a psoas-compartment block and a proximal sciatic nerve block, each using ropivacaine (0.75%, 150 mg), prilocaine (1%, 200 mg), and dexamethasone (4 mg). Surgery was performed with a tourniquet, and drains were removed on postoperative day two. Mobilization began on postoperative day one, with once-daily physiotherapy.

### Pain Management

Analgesia followed the WHO analgesic ladder in both groups. In the ERAS group, patients additionally received a scheduled dose of oxycodone 10 mg twice daily for the first three postoperative days. For both groups, piritramide (3 mg IV) was available as rescue analgesia based on pain severity. Standard oral analgesics included ibuprofen (600 mg), and metamizole (500 mg) administered regularly, with tramadol (100 mg) or oxycodone (10 mg) offered as optional rescue medications if needed.

Post-discharges rehabilitation was self- managed by patients in both groups.

### Isokinetic muscle testing: Biodex^®^ system 3

Objective Muscle strength was evaluated using the Biodex^®^ System 3 Dynamometer (Biodex^®^ Medical Systems, Shirley, NY, USA). This device provides validate measurements of isokinetic knee flexion and extension [26, 27].

Assessments were conducted preoperatively and at 5 days, 4 weeks, 3 months, 6 months, and 12 months postoperatively. Testing followed a standardized Biodex^®^ protocol, including preset parameters and calibration procedures. After a warm-up with five submaximal contractions, patients completed 10 maximal repetitions at 60°/s, followed by a 30-second rest, and then 10 repetitions at 180°/s. Both limbs were tested, starting with the non-operated side.

Measured outcomes included:

- **Peak Torque (in Newton meters**,** Nm)**: Represents the maximum rotational force generated during knee flexion or extension.
- **Total Work (in Joules**,** J)**: Indicates the cumulative energy exerted by the knee joint muscles throughout the range of motion.
- **Power Output (in Watts**,** W)**: Reflects the rate at which muscular work is performed - i.e., how quickly force is applied over time.
- **Range of Motion (ROM) (in degrees)**: Measured during isokinetic knee flexion at 180°/s, this value quantifies the active angular movement capacity of the operated knee.

### Patient-reported outcome measures (PROMs)

To assess subjective recovery and patient satisfaction, a standardized questionnaire was administered both preoperatively and at multiple postoperative time points. This survey evaluated pain perception, functional outcome, and overall quality of life from the patient’s perspective. Items included:

- “In your opinion, was the operation successful?” (Yes/No).
- “Would you choose to undergo the operation again?” (Yes/No).
- “Were your expectations of the operation fulfilled?” (No / Slightly / Moderately / Strongly / Very strongly).
- “How do you feel compared to before the operation?” (Much better / Better / The same / Worse / Much worse).
- “Has your quality of life improved as a result of the operation?” (No / Slightly / Moderately / Strongly / Very strongly).

### Knee injury and osteoarthritis outcome score (KOOS)

KOOS was used to assess subjective recovery across five subscales: pain, symptoms, activities of daily living, sport/recreation, and quality of life - each scored from 0 (worst) to 100 (best) [24].

### Numeric rating scale (NRS)

Pain intensity was recorded using the validated NRS, ranging from 0 (no pain) to 10 (worst imaginable pain) [17].

### Statistical analysis

Data analysis was performed using IBM SPSS Statistics for Windows, Version 27.0 (IBM Corp., Armonk, NY, USA). Continuous variables are reported as mean values with standard deviations (SD), while categorical variables are reported as absolute and relative frequencies (n, %). Between-group comparisons were conducted using the independent samples t-test for normally distributed continuous variables. For non-normally distributed or ordinal continuous variables, the Wilcoxon–Mann–Whitney U test was applied. Binary outcome variables (e.g.,

“Would undergo surgery again”) were analyzed using Fisher’s Exact Test due to small sample sizes and to ensure accurate p-value estimation. Analyses were performed using the available observations at each assessment, and missing values were not imputed. Therefore, the number of participants contributing data could vary between outcomes and follow-up time points (Fig. 1).

A two-sided significance threshold of *p* < 0.05 was used for the individual statistical comparisons. No adjustment for multiple comparisons was performed. Given the number of outcomes, muscle actions, angular velocities, and postoperative assessment time points examined, all individual between-group comparisons should therefore be considered exploratory and hypothesis-generating rather than confirmatory. Accordingly, isolated p-values below 0.05 should be interpreted cautiously because of the increased risk of type I error associated with multiple testing. The analyses did not incorporate a longitudinal mixed-effects or repeated-measures model accounting for within-patient correlation across time; therefore, the individual between-group comparisons should not be interpreted as a formal statistical test of differences in longitudinal recovery trajectories.

## Results

A total of 62 patients were initially enrolled. Four patients withdrew before postoperative data acquisition, leaving 58 patients for analysis (31 ERAS and 27 conventional). During follow-up, some participants declined further study visits, primarily because of the travel distance and time required for repeated assessments. The number of participants contributing data therefore varied across follow-up time points, as shown in Fig. 1. There were no significant differences in baseline demographic or clinical characteristics between the groups (see Table 1). The mean age was 67.94 ± 8.46 years in the ERAS group and 66.74 ± 8.05 years in the conventional group (*p* = 0.585). Body mass index (BMI) was also comparable, with values of 31.89 ± 5.61 and 31.09 ± 4.65, respectively (*p* = 0.566).

No postoperative wound complications, surgical-site or periprosthetic joint infections, clinically diagnosed thromboembolic events, or other clinically relevant adverse events occurred in either group. No 30-day hospital readmissions or revision surgeries were recorded during the one-year follow-up period.

Prior to surgery, all patients reported impaired knee function. Overall, 48% of participants rated their function as “impaired” (50% in the conventional group and 46% in the ERAS group), while 52% described it as “severely impaired” (50% Conventional, 54% ERAS).

### Pain (NRS)

Pain levels, assessed using the Numeric Rating Scale (NRS), declined substantially postoperatively in both groups (see Table 1). Preoperatively, both groups reported a median pain score of 6. At 4 weeks, pain decreased to a median of 2 in the ERAS group and 3 in the conventional group. Although not statistically significant, the ERAS group exhibited a trend toward faster early pain relief. Pain scores remained low across all follow-ups points up to one year (median ≤ 2), without significant intergroup differences (all *p* > 0.05).

### Self-perceived health

Self-reported health status improved continuously over time (see Table 1). At baseline, median values were slightly higher in the conventional group (60) than in the ERAS group (50), though the difference was not significant (*p* = 0.182). At 4 weeks, median values improved to 70 (ERAS) and 75 (Conventional). By 6 months, both groups reached a median score of 80, which remained stable through the follow-up after 12 months (ERAS: 80; Conventional: 82.5). Differences between groups were not statistically significant at any time point (all *p* > 0.05).

**Table 1:** Comparison of age, BMI, Numeric Rating Scale (NRS) for pain, and self-reported current health status between ERAS and conventional groups at multiple postoperative time points. p-values of Age and BMI were calculated using the independent samples t-test. p-values of NRS and current health status were calculated using the Wilcoxon Mann–Whitney U test

	*N*	total	ERAS (*n* = 31)	Conventional (*n* = 27)	*p*-Score
Age	58	67.4 (SD 8.22)	67.9 (SD 8.46)	66.7 (SD 8.05)	0,585
BMI	56	31.5 (SD 5.14)	31.9 (SD 5.61)	31.1 (SD 4.65)	0,566
Pain measured using the Numeric Rating Scale (NRS)					
Pre-surgery	40	6 (IQR 5–7)	6 (IQR 5-7.25)	6 (IQR 5.62-7)	1,000
4 weeks postoperatively	49	2.5 (IQR 2–4)	2 (IQR 2-3.75)	3 (IQR 2–4)	0,360
3 months postoperatively	38	2 (IQR 1.25–3.75)	2 (IQR 1-2.75)	2.25 (IQR 2–4)	0,249
6 months postoperatively	44	2 (IQR 1-3.25)	2 (IQR 1–3)	2 (IQR 1–4)	0,738
1 year postoperatively	39	2 (IQR 1–4)	2 (IQR 1-4.25)	2 (IQR 1-2.5)	0,264
Self-reported current health status					
Pre-surgery	58	60 (IQR 50–65)	50 (IQR 40–65)	60 (IQR 50-72.5)	0,182
4 weeks postoperatively	53	75 (IQR 60–80)	70 (IQR 59–80)	75 (IQR 65-81.25)	0,322
3 months postoperatively	43	75 (IQR 60-82.5)	70 (IQR 60–75)	80 (IQR 66.25–88.75)	0,103
6 months postoperatively	47	80 (IQR 71.5–86.5)	80 (IQR 65-82.5)	80 (IQR 75–90)	0,232
1 year postoperatively	40	80 (IQR 68.75-90)	80 (IQR 57.5-81.25)	82.5 (IQR 70–95)	0,107

### Range of motion (ROM)

**Fig. 2:**
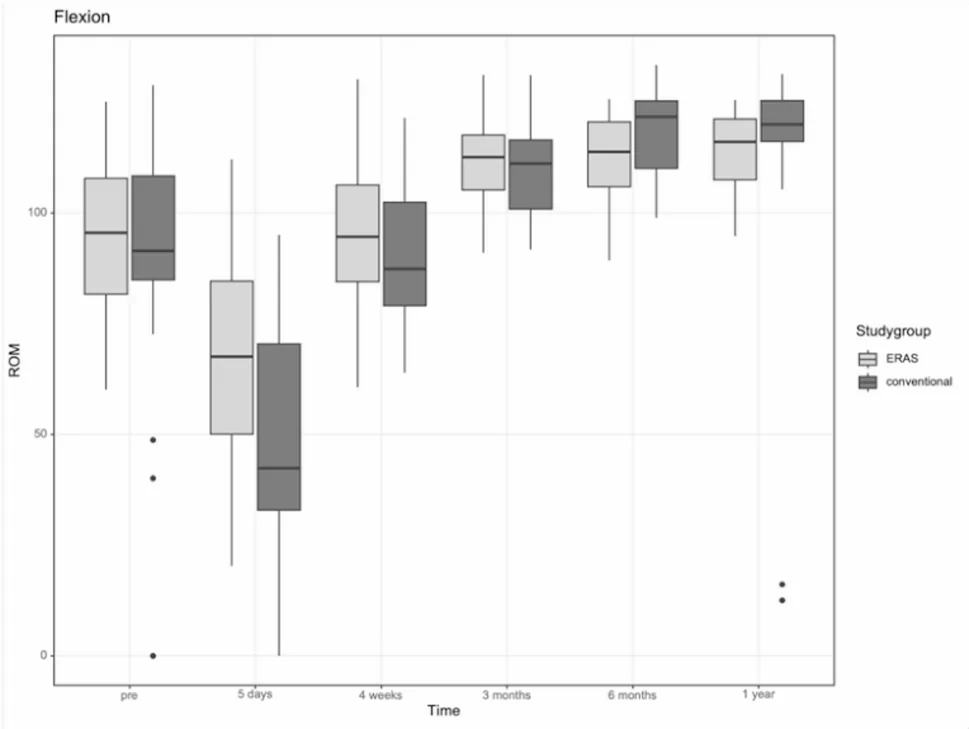
Range of motion (ROM) during flexion at 180°/s comparing the Enhanced Recovery After Surgery (ERAS) group and the conventional group. The x-axis represents the time points of evaluation: Preoperative, 5 days, 4 weeks, 3 months, 6 months, and 1 year postoperatively. The y-axis shows the range of motion (ROM) in degrees (°). p-values were calculated using the Wilcoxon Mann–Whitney U test. Asterisks (*) indicate statistically significant differences with *p* < 0.05

Range of motion (ROM) showed continuous improvement over time in both groups (see Fig. 2). Preoperative ROM values were comparable. At 5 days postoperatively, the ERAS group demonstrated significantly greater ROM (median 67.55° vs. 42.3°, *p* = 0.031), indicating early mobility benefits of the accelerated protocol. This advantage persisted at 4 weeks and 3 months, although differences were not statistically significant. By 6 months, both groups achieved excellent ROM with medians exceeding 110°. Notably, the ERAS group showed consistently narrower interquartile ranges, suggesting a more uniform recovery trajectory.

### Peak torque (Nm)

**Fig. 3:**
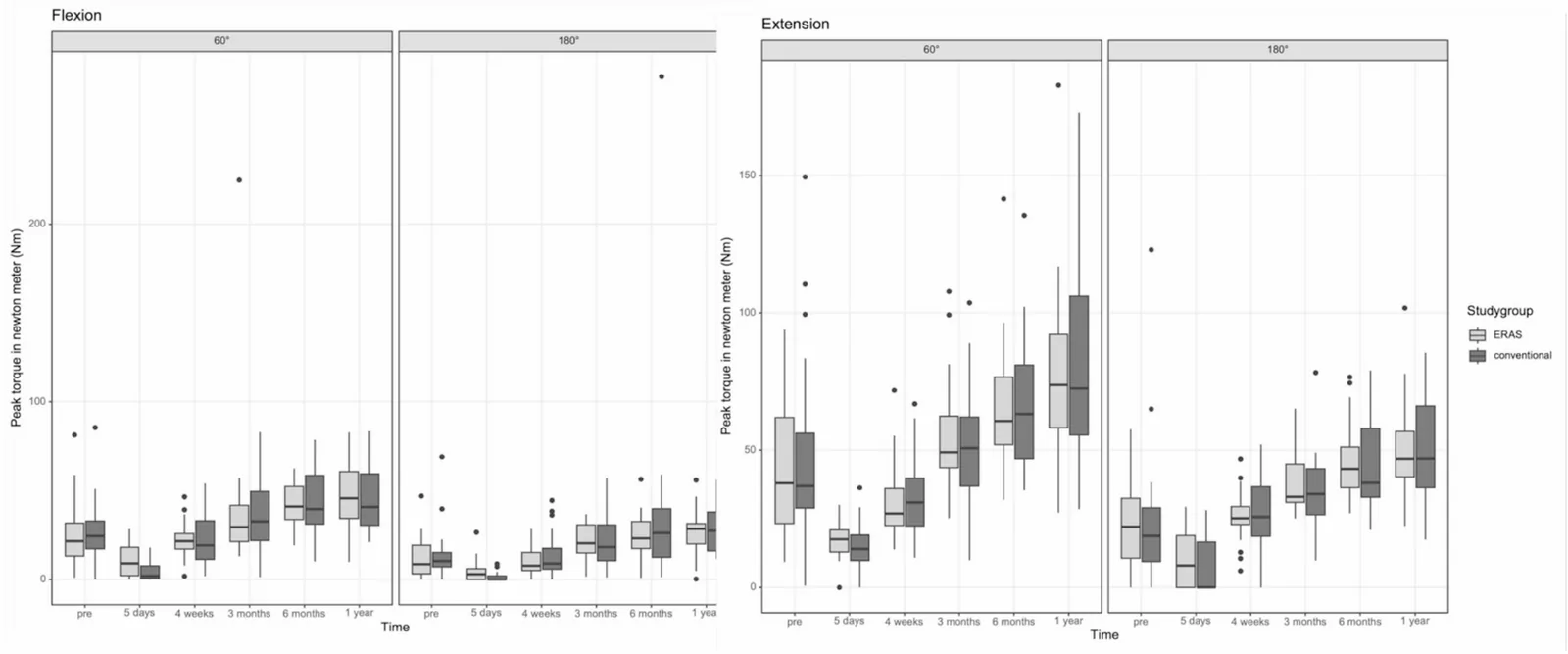
*Peak torque values of the operated knee during flexion (a) and extension (b) at two angular velocities (60°/s and 180°/s). The x-axis shows the time points of evaluation for the Enhanced Recovery After Surgery (ERAS) group versus the conventional group: preoperatively*,* and at 5 days*,* 4 weeks*,* 3 months*,* 6 months*,* and 1 year postoperatively. The y-axis represents peak torque in newton meters (Nm). p-values were calculated using the Wilcoxon Mann–Whitney U test.* Asterisks (*) indicate statistically significant differences with *p* < 0.05

Isokinetic testing demonstrated progressive improvements in both groups over time. At 5 days, the ERAS group exhibited significantly higher peak torque in knee flexion at 60°/s (*p* = 0.012), whereas the difference at 180°/s did not reach statistical significance (*p* = 0.053). These findings suggest an early advantage in isokinetic muscle performance in the ERAS group. Similar early advantages were observed for power and total work. By 4 weeks, group differences had diminished, with comparable recovery patterns across all strength measures. Improvements continued at 3 and 6 months, and by 1 year, both groups had achieved near-complete recovery, with no significant between-group differences in peak torque (see Fig. 3a and b). The ERAS group demonstrated more stable interquartile ranges over time, particularly at 180°/s.

### Total work (J)

**Fig. 4:**
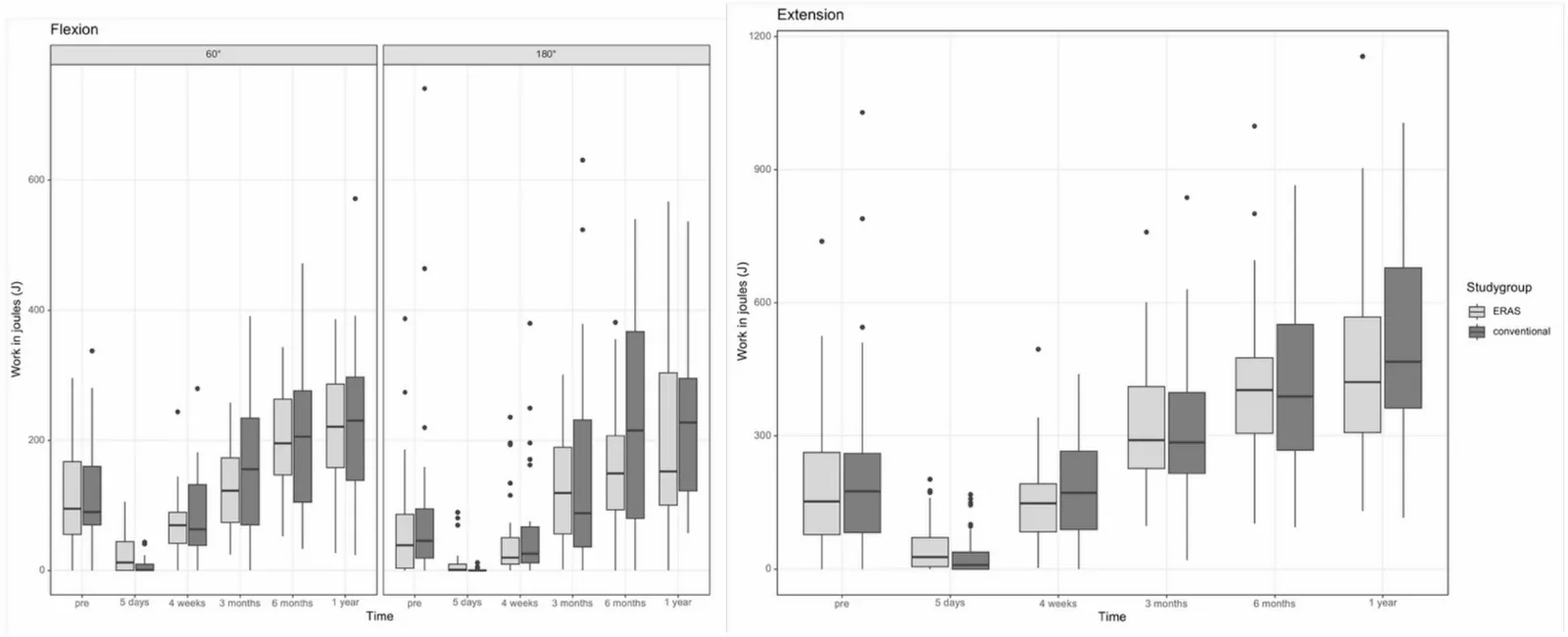
*Total work of the operated knee during flexion (a) measured at two angular velocities (60°/s and 180°/s) and during extension at 60°/s (b). The x-axis displays the evaluation time points for the Enhanced Recovery After Surgery (ERAS) group versus the conventional group: preoperatively*,* and at 5 days*,* 4 weeks*,* 3 months*,* 6 months*,* and 1 year postoperatively. The y-axis represents work in joules (J). p-values were calculated using the Wilcoxon Mann–Whitney U test.* Asterisks (*) indicate statistically significant differences with *p* < 0.05

Total work output (in joules) increased over time in both groups (see Fig. 4a and b). At 5 days, the ERAS group achieved significantly higher work during knee flexion at 60°/s and 180°/s (*p* = 0.022 and *p* = 0.038). During knee extension at 60°/s, the ERAS group showed a trend toward higher work output, without reaching statistical significance at 5 days, 3 months, or 6 months after surgery.

At 4 weeks and 3 months, both groups demonstrated comparable improvements, although the ERAS group exhibited slightly more consistent performance. By 6 months, work values were high and did not differ significantly between groups. Although the conventional group showed slightly higher values in some parameters at later time points, these differences were not statistically significant.

### Power (W)

**Fig. 5:**
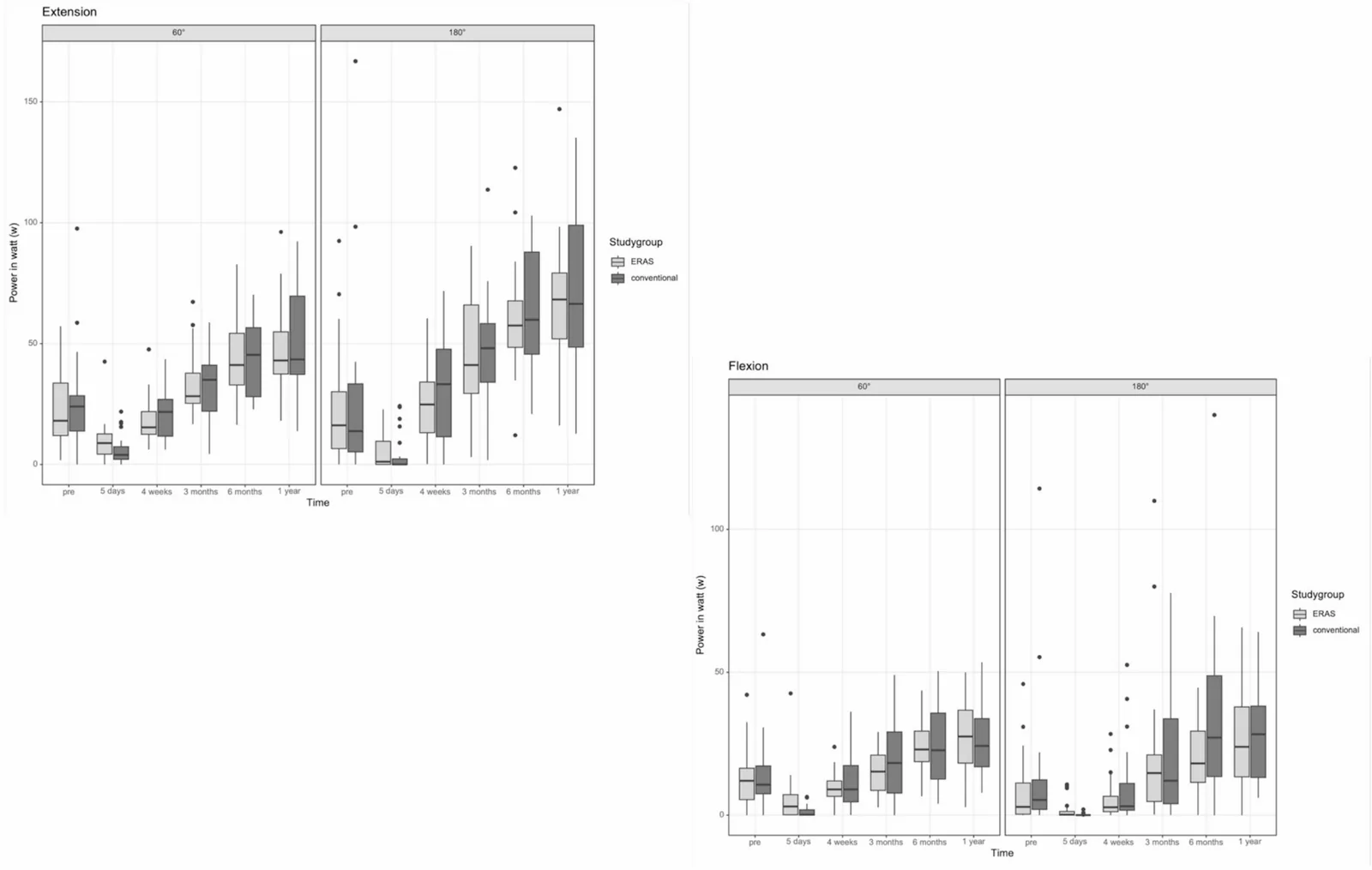
*Power output of the operated knee during flexion (a) and extension (b) measured at two angular velocities (60°/s and 180°/s). The x-axis shows the time points of evaluation for the Enhanced Recovery After Surgery (ERAS) group versus the conventional group: preoperatively*,* and at 5 days*,* 4 weeks*,* 3 months*,* 6 months*,* and 1 year postoperatively. The y-axis represents power in watts (W).p-values were calculated using the Wilcoxon Mann–Whitney U test.* Asterisks (*) indicate statistically significant differences with *p* < 0.05

Power (in watts) improved steadily in both groups (see Fig. 5a and b), with comparable preoperative values. At 5 days postoperatively, the ERAS group showed significantly higher power in flexion at both 60°/s and 180°/s (*p* = 0.007 and *p* = 0.011), as well as in extension at 60°/s (*p* = 0.036).

By 4 weeks, power had increased in both groups, with the conventional group showing a slight, non-significant advantage in extension at both speeds. At 3 and 6 months, both groups achieved high performance levels across all settings. Early postoperative advantages in the ERAS group diminished over time, resulting in comparable outcomes between groups.

### Knee Injury and Osteoarthritis Outcome Score (KOOS)

**Fig. 6:**
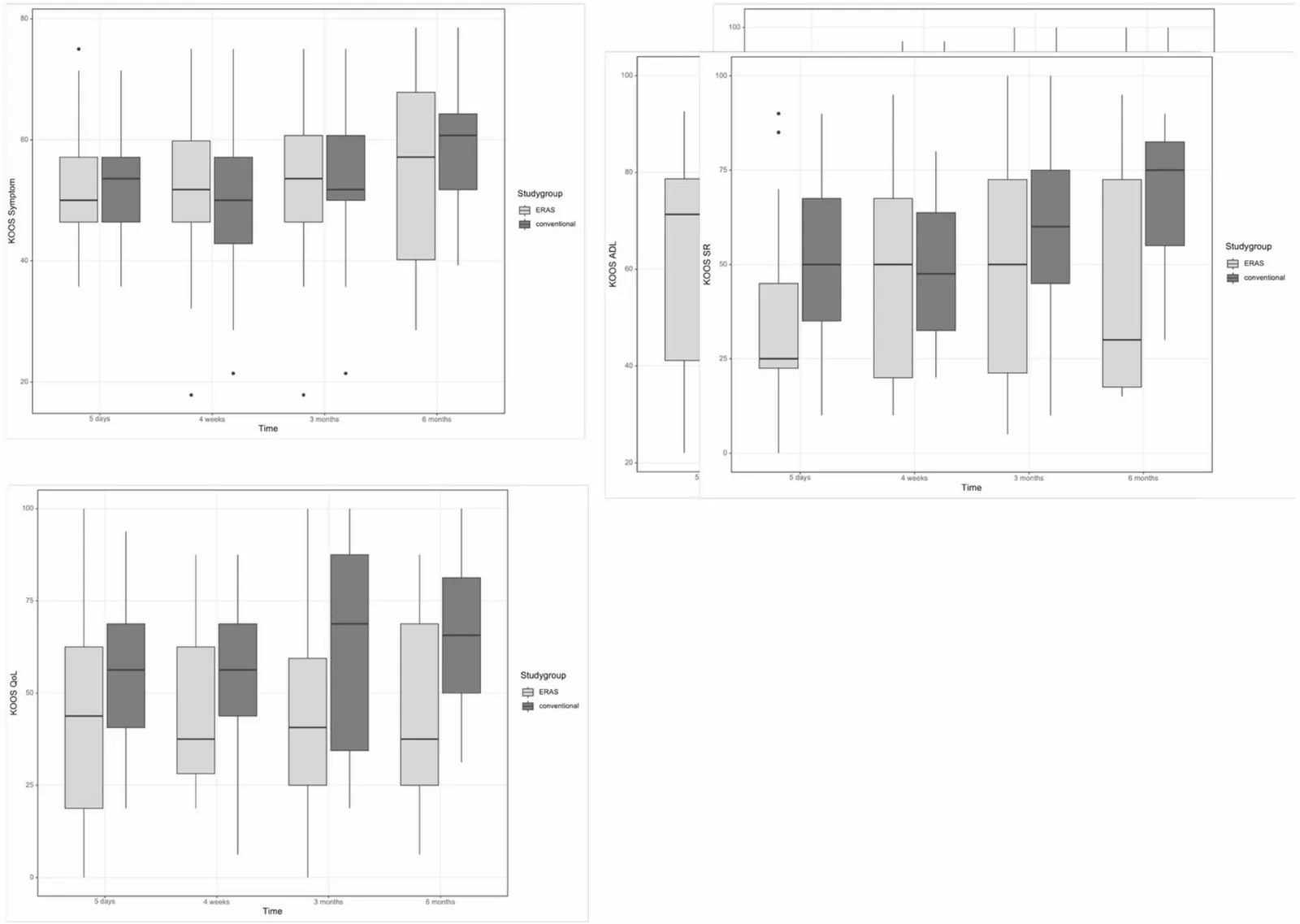
*Boxplot visualization of Knee Injury and Osteoarthritis Outcome Score (KOOS) subscale scores across five postoperative time points in the Enhanced Recovery After Surgery (ERAS) and conventional groups. The KOOS domains shown include*

KOOS subscales were comparable preoperatively (see Figs. 6a − 6e). At 4 weeks, the ERAS group showed more rapid improvement, particularly regarding “Symptoms” and “Activities of Daily Living” subscales. These early advantages persisted at 3 and 6 months, with narrower interquartile ranges (IQRs) reflecting more consistent recovery in the ERAS group.

At 1 year, both groups demonstrated strong functional outcomes; however, the ERAS group maintained its early gains in symptom control and daily function. Although the conventional group showed slightly higher absolute scores in some subscales at later time points, ERAS patients exhibited faster functional recovery and more predictable outcome patterns.

- *Symptoms (S)*.
- *Pain (P)*.
- *Activities of daily living (ADL)*.
- *Sport/recreation (SR)*.
- *Quality of life (QoL)*.

*Evaluations were performed preoperatively and at 4 weeks*,* 3 months*,* 6 months*,* and 1 year postoperatively. The y-axis represents KOOS scores ranging from 0 (worst) to 100 (best). p-values were calculated using the Wilcoxon Mann–Whitney U test.* Asterisks (*) indicate statistically significant differences with *p* < 0.05.

### PROMS

At 4 weeks, all patients considered the surgery successful and indicated that they would undergo the procedure again. More than 85% reported feeling “better” or “much better” than before surgery, and over 60% reported a strong or very strong improvement in quality of life. Expectations were rated as “strongly” or “very strongly” fulfilled by most patients.

At 3 months, satisfaction remained high; however, three patients in the conventional group no longer considered the surgery successful. A significant between-group difference in expectation fulfilment was observed (*p* = 0.019), favouring the ERAS group.

By 6 months, more than 90% of participants continued to rate the operation as successful, with approximately 50% reporting strong or very strong improvements in quality of life. At 1 year, 92% still considered the operation successful, and 95% indicated that they would choose to undergo the procedure again. Overall, expectations remained fulfilled, and more than 90% of patients reported feeling better or much better than preoperatively.

Apart from the significant difference in expectation fulfilment at 3 months, no further statistically significant between-group differences in PROMs were observed (all *p* > 0.05; see Table 2).

**Table 2:** Patient-reported outcome measures (PROMs) at 4 weeks, 3 months, 6 months, and 1 year following total knee arthroplasty in the ERAS and conventional groups. Data are presented as absolute numbers and percentages. Binary outcome variables (e.g., “Would undergo surgery again”) were analysed using Fisher’s Exact Test; ordinal responses (e.g., “Expectation fulfilled”) were compared using the Mann–Whitney U test. *p < 0.05 was considered statistically significant

Variables	total	ERAS	Conventional		*p*-Score
4 Weeks					
In your opinion, was the operation successful?					-
yes	52/52 (100%)	29/29 (100%)		23/23 (100%)	
no	0/52 (0%)	0/29 (0%)		0/23 (0%)	
Would you choose to undergo the operation again?					-
yes	51/51 (100%)	30/30 (100%)		21/21 (100%)	
no	0/51 (0%)	0/30 (0%)		0/21 (0%)	
Were your expectations of the operation fulfilled?					0,837
no	2/52 (4%)	0/30 (0%)		2/22 (9%)	
slightly	0//52 (0%)	0/30 (0%)		0/22 (0%)	
moderately	6/52 (12%)	4/30 (13%)		2/22(9%)	
strongly	28/52 (54%)	17/30 (57%)		11/22 (50%)	
very strongly	16/52 (31%)	9/30 (30%)		7/22 (32%)	
How do you feel compared to before the operation?					0,564
much better	21/53 (40%)	13/30 (43%)		8/23 (35%)	
better	24/53 (45%)	13/30 (43%)		11/23 (48%)	
the same	6/53 (11%)	2/30 (7%)		4/23 (17%)	
worse	2/53 (4%)	2/30 (7%)		0/23 (0%)	
much worse	0/53 (0%)	0/30 (0%)		0/23 (0%)	
Has your quality of life improved as a result of the operation?					0,512
no	4/53 (8%)	2/30 (7%)		2/23 (9%)	
slightly	3/53 (6%)	1/30 (3%)		2/23 (9%)	
moderately	12/*53* (23%)	7/30 (23%)		5/23 (22%)	
strongly	28/53 (53%)	16/30 (53%)		12/23 (52%)	
very strongly	6/53 (11%)	4/30 (13%)		2/23 (9%)	
3 Months					
In your opinion, was the operation successful?					0,234
yes	41/44 (93%)	21/21 (100%)		20/23 (87%)	
no	3/44 (7%)	0/21 (0%)		3/23 (13%)	
Would you choose to undergo the operation again?					0,488
yes	41/43 (95%)	21/21(100%)		20/22 (91%)	
no	2/43 (5%)	0/21 (0%)		2/22 (9%)	
Were your expectations of the operation fulfilled?					**0**,019*
no	1/44 (2%)	0/21 (0%)		1/23 (4%)	
slightly	4/44 (9%)	1/21 (5%)		3/23 (13%)	
moderately	1/44 (2%)	0/21 (0%)		1/23 (4%)	
strongly	29/44 (66%)	13/21(62%)		16/23 (70%)	
very strongly	9/44 (20%)	7/21 (33%)		2/23 (9%)	
How do you feel compared to before the operation?					0,153
much better	16/44 (36%)	9/21 (43%)		7/23 (30%)	
better	24/44 (55%)	12/21 (57%)		12/23 (52%)	
the same	3/44 (7%)	0/21 (0%)		3/23 (13%)	
worse	0/44 (0%)	0/21 (0%)		0/23 (0%)	
much worse	1/44 (2%)	0/21 (0%)		1/23 (4%)	
Has your quality of life improved as a result of the operation?					0,727
no	1/43 (2%)	0/21 (0%)		1/22 (5%)	
slightly	3/43 (7%)	1/21 (5%)		2/22 (9%)	
moderately	8/43 (19%)	5/21 (24%)		3/22 (14%)	
strongly	24/43 (56%)	11/21 (52%)		13/22 (59%)	
very strongly	7/43 (16%)	4/21 (19%)		3/22 (14%)	
6 Months					
In your opinion, was the operation successful?					0,659
yes	41/46 (89%)	19/22 (86%)		22/24 (92%)	
no	5/46 (11%)	3/22 (14%)		2/24 (8%)	
Would you choose to undergo the operation again?					1,000
yes	42/46 (91%)	21/23 (91%)		21/23 (91%)	
no	4/46 (9%)	2/23 (9%)		2/23 (9%)	
Were your expectations of the operation fulfilled?					0,511
no	4/47 (9%)	2/23 (9%)		2/24 (8%)	
slightly	5/47 (11%)	3/23 (13%)		2/24 (8%)	
moderately	3/47 (6%)	1/23 (4%)		2/24 (8%)	
strongly	24/47 (51%)	13/23 (57%)		11/24 (46%)	
very strongly	11/47 (23%)	4/23 (17%)		7/24 (29%)	
How do you feel compared to before the operation?					0,549
much better	25/47 (53%)	13/23 (56%)		12/24 (50%)	
better	17/47 (36%)	8/23 (35%)		9/24 (38%)	
the same	4/47 (9%)	1/23 (4%)		3/24 (12%)	
worse	1/47 (2%)	1/23 (4%)		0/24 (0%)	
much worse	0/47 (0%)	0/23 (0%)		0/24 (0%)	
Has your quality of life improved as a result of the operation?					0,777
no	3/45 (7%)	2/21 (10%)		1/24 (4%)	
slightly	4/45 (9%)	1/21 (5%)		3/24 (12%)	
moderately	7/45 (16%)	3/21 (14%)		4/24 (17%)	
strongly	23/45 (51%)	11/21 (52%)		12/24 (50%)	
very strongly	8/45 (18%)	4/21 (19%)		4/24 (17%)	
1 Year					
In your opinion, was the operation successful?					1,000
yes	37/40 (92%)	18/20 (90%)		19/20 (95%)	
no	3/40 (8%)	2/20 (10%)		1/20 (5%)	
Would you choose to undergo the operation again?					1,000
yes	38/40 (95%)	19/20 (95%)		19/20 (95%)	
no	2/40 (5%)	1/20 (5%)		1(20 (5%)	
Were your expectations of the operation fulfilled?					0,870
no	2/40 (5%)	2/20 (10%)		0 (0%)	
slightly	3/40 (8%)	2/20 (10%)		1/20 (5%)	
moderately	6/40 (15%)	2/20 (10%)		4/20 (20%)	
strongly	22/40 (55%)	9/20 (45%)		13/20 (65%)	
very strongly	7/40 (18%)	5/20 (25%)		2/20 (10%)	
How do you feel compared to before the operation?					0,563
much better	22/40 (55%)	10/20 (50%)		12/20 (60%)	
better	14/40 (35%)	8/20 (40%)		6/20 (30%)	
the same	3/40 (8%)	1/20 (5%)		2/20 (10%)	
worse	0/40 (0%)	0/20 (0%)		0/20 (0%)	
Much worse	1/40 (2%)	1/20 (5%)		0/20 (0%)	
Has your quality of life improved as a result of the operation?					0,602
no	1/40 (2%)	1/20 (5%)		0/20 (0%)	
slightly	4/40 (10%)	2/20 (10%)		2/20 (10%)	
moderately	6/40 (15%)	4/20 (20%)		2/20 (10%)	
strongly	22/40 (55%)	9/20 (45%)		13/20 (65%)	
very strongly	7/40 (18%)	4/20 (20%)		3/20 (15%)	

## Discussion

In this prospective, single-blinded randomized controlled trial, we evaluated whether a modified ERAS protocol improves recovery following primary total knee arthroplasty compared with conventional perioperative care. Overall, our findings indicate that ERAS primarily enhances early postoperative recovery across objective strength measures and patient-reported outcomes, while differences between groups diminish over time.

These findings are consistent with a growing body of evidence demonstrating that ERAS protocols facilitate accelerated functional recovery, reduce hospital stay, and improve patient satisfaction [2, 3, 6, 28, 29]. However, not all studies have shown sustained superiority of ERAS beyond the early postoperative phase. For example, Wei et al. reported improved early functional outcomes following ERAS in TKA but similarly observed convergence of outcomes at later follow-up intervals, suggesting that long-term recovery may be less influenced by perioperative pathways alone [30].

The superior early postoperative muscle function observed in the ERAS group - reflected by higher peak torque, total work, and power output at day 5 - may be related to several synergistic mechanisms. Early mobilization, reduced surgical stress response, optimized multimodal analgesia, and structured physiotherapy may facilitate improved neuromuscular activation and reduce early postoperative quadriceps inhibition [15, 16]. Comparable findings have been reported in total hip arthroplasty, where ERAS protocols were associated with enhanced early neuromuscular recovery [19].

Despite these early advantages, group differences diminished by 3 to 6 months. Several factors may explain this convergence. First, patients in conventional rehabilitation pathways may “catch up” over time, achieving similar functional endpoints. Second, biological recovery processes, including muscle regeneration and joint adaptation, may increasingly influence mid- and long-term outcomes, thereby attenuating the relative impact of perioperative strategies. Third, patient-related factors such as adherence, activity level, and rehabilitation intensity may outweigh initial perioperative differences in later phases. Similar patterns of early benefit followed by convergence have been reported in previous ERAS studies and meta-analyses [28, 31, 32].

Total work, reflecting cumulative muscular endurance, also favoured the ERAS group in the early recovery phase. This supports previous findings by Wainwright et al., who demonstrated functional improvements in ERAS patients as early as the inpatient setting [7]. Our results underline the importance of early mobilization not only for strength but also for endurance recovery. However, the absence of long-term differences suggests that endurance capacity ultimately becomes comparable across rehabilitation strategies.

Power output, as an indicator of dynamic neuromuscular performance, was likewise higher in the ERAS group early after surgery. Previous studies have linked higher postoperative power values with improved functional outcomes such as gait speed and stair-climbing ability [33–35]. Thus, the enhanced power output observed in our cohort may reflect more efficient early neuromuscular activation and faster return to functional independence. Early improvements in range of motion further support the role of ERAS in facilitating rapid functional recovery. Enhanced mobilization likely reduces joint stiffness and promotes more favourable recovery trajectories, as also described by Wainwright et al. [20, 29, 36]. However, these early differences diminished during subsequent follow-up.

Patient-reported outcomes closely paralleled objective findings. Faster early pain reduction in the ERAS group is likely attributable to multimodal analgesia and reduced opioid use, as highlighted in previous studies [36]. Although these differences were not sustained long-term, early postoperative pain experience is clinically relevant, as it influences patient satisfaction, mobilization, and overall recovery perception.

The observed difference in expectation fulfilment at 3 months suggests that ERAS may better align early recovery with patient expectations rather than simply improving outcomes per se [22]. This may be explained by faster functional gains, earlier independence, and structured perioperative education [36]. Neuprez et al. identified expectation alignment as a key determinant of postoperative satisfaction, particularly in active patient populations [22]. The absence of sustained differences at later time points indicates that long-term satisfaction is likely multifactorial and less dependent on perioperative pathways alone.

KOOS subscales indicated faster early improvements in symptoms and activities of daily living in the ERAS group. However, these differences diminished during follow-up, and long-term KOOS outcomes were comparable between groups. Comparable improvements in KOOS outcomes have been reported in ERAS-based rehabilitation strategies [21].

Taken together, our findings suggest that the principal benefit of the modified ERAS protocol was observed during the early postoperative phase after TKA. Between-group differences diminished during subsequent follow-up, resulting in largely comparable mid-term objective and patient-reported outcomes. Thus, the findings support accelerated early recovery with ERAS rather than sustained superiority over conventional perioperative care [7, 28, 29, 32]. By integrating objective biomechanical performance measures with validated patient-reported outcomes, this study provides a comprehensive assessment of recovery, encompassing both physical function and patient-perceived outcomes.

### Strengths and limitations

Strengths of this study include its prospective, randomized controlled, and single-blinded design, which represents the gold standard in clinical research and reduces selection and detection bias, thereby enhancing internal validity.

A key strength was the comprehensive assessment of outcomes, combining objective measures such as peak torque, total work, and power output with validated subjective instruments, including the KOOS, NRS, and multiple PROMs capturing patient satisfaction, perceived recovery, and health-related quality of life. This dual approach allowed a more nuanced understanding of how biomechanical recovery aligns with patient perception.

Furthermore, the study included five follow-up time points from the early postoperative phase (day 5) to one year, providing a detailed view of recovery over time. The inclusion of isokinetic testing – a precise and reproducible method – enabled the detection of subtle changes in knee function not always captured by standard clinical assessments.

Nevertheless, several limitations must be acknowledged. The relatively small sample size (*n* = 58) may limit statistical power, particularly for subgroup analyses or the detection of subtle effects in later phases. Participant numbers decreased during follow-up, primarily because some patients declined further assessments due to the travel distance and time required for repeated study visits. Importantly, no loss to follow-up was attributable to revision surgery, postoperative complications, or death. Thus, the reduction in participant numbers was predominantly related to logistical reasons rather than adverse clinical outcomes, although the smaller sample size at later follow-up time points should be considered when interpreting these findings. Although patients were not informed of their treatment allocation and the ERAS and conventional-care groups were accommodated on separate wards, complete blinding could not be guaranteed because of visible differences in postoperative management, particularly the use of suction drainage in the conventional-care group. Treatment allocation may therefore have been inferred, representing a potential source of bias, particularly for subjective outcomes such as PROMs and expectation ratings.

An additional limitation is that the two treatment pathways differed in several perioperative components, including the use of suction drainage. No drain was used in the ERAS group, whereas suction drainage was maintained until postoperative day 2 in the conventional-care group. Drainage itself may influence early mobilization, discomfort, wound management, and potentially early functional recovery and therefore represents a potential confounding factor. Consequently, the observed early differences cannot be attributed to a single component of the ERAS protocol but should be interpreted as the effect of the multimodal pathway as a whole.

It should also be noted that while isokinetic testing provides granular data on muscle performance, it does not fully reflect complex real-world functions such as gait, stair climbing, or return to occupational or athletic activities. Moreover, the study was conducted at a single center, which may limit generalizability. Institutional differences in rehabilitation practices and surgical techniques could affect the external validity of the findings.

The early functional outcome of TKA is associated with implant positioning. A detailed analysis of component positioning could not be performed due to radiation protection regulations in the country where the study was conducted. In particular, rotational component alignment was not assessed by postoperative CT, and preoperative tibial varus was not specifically considered when determining tibial component rotation. Although a potential influence of individual tibial anatomy and component rotation on functional outcomes cannot be excluded, randomization likely resulted in a comparable distribution of potential rotational deviations between the two groups. All procedures were carried out using imageless navigation and mechanical alignment. In all patients, the postoperative mechanical axis, as confirmed by the final measurement obtained with the imageless navigation system, was 0° ± 3°.

An important statistical limitation is the use of multiple unadjusted between-group comparisons, increasing the risk of type I error. Individual significant findings should therefore be considered exploratory. In addition, repeated measurements within patients were not accounted for using longitudinal mixed-effects models. Accordingly, emphasis should be placed on the overall pattern of early ERAS benefits followed by convergence between groups over time. Future studies should apply prespecified longitudinal models and appropriate multiplicity correction.

## Conclusion

In summary, this randomized controlled trial indicates that a modified ERAS protocol primarily provides measurable benefits during the early postoperative recovery phase following primary total knee arthroplasty, particularly regarding isokinetic muscle performance and functional recovery. These between-group differences diminished during subsequent follow-up, with largely comparable clinical and patient-reported outcomes between the groups at the mid-term assessments.

By integrating objective biomechanical data with validated patient-reported outcome measures, the present findings suggest that the main benefit of the modified ERAS pathway lies in accelerating early recovery rather than providing sustained superiority over conventional perioperative care.

Future studies with larger patient cohorts are warranted to validate these findings and further evaluate the long-term effects of ERAS following TKA.

## Supplementary Information

Below is the link to the electronic supplementary material.

Supplementary Material 1

## Data Availability

On request, data is available at the authors’ institution.

## Abbreviations

ERAS: Enhanced Recovery After Surgery
IQR: Interquartile Range
Md: Median
p: p-value
SD: Standard deviation
TKA: Total knee arthroplasty

## References

[1] Kehlet, H. & Wilmore, D. W. Evidence-based surgical care and the evolution of fast-track surgery. *Ann Surg.* **248**, 189–98 10.1097/SLA.0b013e31817f2c1a (2008). 18650627

[2] Zhang, Q., Chen, Y., Li, Y., Liu, R., Rai, S., Li, J. & Hong, P. Enhanced recovery after surgery in patients after hipand knee arthroplasty: a systematic review and meta-analysiss. *Postgrad Med J* **100**, 159–173 10.1093/postmj/qgad125 (2024). 38134323

[3] Ishaku, Z., Koshy, D. I. & Adamu Bala, M. Enhanced Recovery After Surgery(ERAS) Pathways in Elective Total Joint Arthroplasty. *Cureus* **17**, e91481 10.7759/cureus.91481 (2025). PMC12412062 40917910

[4] den Hertog, A., Gliesche, K., Timm, J., Mühlbauer, B. & Zebrowski, S. Pathway-controlled fast-trackrehabilitation after total knee arthroplasty: a randomized prospective clinical study evaluating the recovery pattern, drug consumption, and length of stay. *Arch Orthop Trauma Surg* **132**, 1153–63 10.1007/s00402-012-1528-1 (2012). PMC3400756 22643801

[5] Larsen, K., Hansen, T. B., Thomsen, P. B., ChristiansenT & Søballe, K. Cost-effectiveness of accelerated perioperative care and rehabilitation after total hip and knee arthroplasty. *J Bone Joint Surg Am.* **91**, 761–72 10.2106/JBJS.G.01472 (2009). 19339559

[6] Kumar, L., Kumar, A. H., Grant, S. A. & Gadsden, J. Updates in Enhanced Recovery Pathways forTotal Knee Arthroplasty. *Anesthesiol Clin* **36**, 375–386 10.1016/j.anclin.2018.04.007 (2018). 30092935

[7] Wainwright, T. W., Gill, M., McDonald, D. A., MiddletonRG, Reed, M., Sahota, O., Yates, P. & Ljungqvist, O. Consensus statement for perioperative care in total hip replacement and total knee replacement surgery:Enhanced Recovery After Surgery (ERAS®) Society recommendations. *Acta Orthop* **91**, 3–19 10.1080/17453674.2019.1683790 (2020). PMC7006728 31663402

[8] Goetz, J., Maderbacher, G., Gerg, A., Leiss, F., Dullien, S., Zeman, F., Meyer, M., Reinhard, J., Grifka, J. & Greimel, F. Isokinetic kneemuscle strength comparison after enhanced recovery after surgery (ERAS) versus conventional setup in total knee arthroplasty (TKA): a single blindedprospective randomized study. *J Exp Orthop* **10**, 44 10.1186/s40634-023-00604-0 (2023). PMC10105813 37060486

[9] Baker, P. N., van der Meulen, J. H., Lewsey, J. & Gregg, P. J. The role of pain and function in determining patient satisfaction after total knee replacement. Data from the National Joint Registryfor England and Wales. *J Bone Joint Surg Br* **89**, 893–900 10.1302/0301-620X.89B7.19091 (2007). 17673581

[10] Kurtz, S., Ong, K., Lau, E., Mowat, F. & Halpern, M. Projections of primary and revision hip and knee arthroplasty in the UnitedStates from 2005 to 2030. *J Bone Joint Surg Am* **89**, 780–5 10.2106/JBJS.F.00222 (2007). 17403800

[11] Stevens-Lapsley, J. E., Balter, J. E., Wolfe, P., Eckhoff, D. G. & Kohrt, W. M. Early neuromuscular electrical stimulation toimprove quadriceps muscle strength after total knee arthroplasty: a randomized controlled trial. *Phys Ther* **92**, 210–26 10.2522/ptj.20110124 (2012). PMC3269772 22095207

[12] Bakırhan, S., Unver, B., Elibol, N. & Karatosun, V. Fear of movement and other associated factors in older patients with total knee arthroplasty. *Ir J Med Sci* **192**, 2217–2222 10.1007/s11845-022-03214-4 (2023). 36445627

[13] Aftab, S., Ali, H. N., Saeed, B., Sarwar, S., Dawood, M. H. & Pervez, M. Early Physiotherapy for Post-Total Knee Arthroplasty Recovery:A Systematic Review of Randomized Controlled Trials on Quality of Life, Pain, and Range of Motion Outcomes. *Musculoskeletal Care* **23**, e70158 10.1002/msc.70158 (2025). 40635148

[14] Guerra, M. L., Singh, P. J. & Taylor, N. F. Early mobilizationof patients who have had a hip or knee joint replacement reduces length of stay in hospital: a systematic review. *Clin Rehabil* **29**, 844–54 10.1177/0269215514558641 (2015). 25452634

[15] Lisi, C., Caspani, P., Bruggi, M., Carlisi, E., Scolè, D., Benazzo, F. & Dalla Toffola, E. Early rehabilitation afterelective total knee arthroplasty. *Acta Biomed* **88**, 56–61 10.23750/abm.v88i4-S.5154 (2017). PMC6357664 29083354

[16] Humphrey, T. J., Salimy, M. S., Jancuska, J. M., Egan, C. R., Melnic, C. M., Alpaugh, K. & Bedair, H. S. Sarcopenia is an independent risk factor for failure to achieve the 1-year MCID of the KOOS, JR andPROMIS PF-SF10a after TKA. *Knee.* **42**, 64–72 10.1016/j.knee.2023.02.018 (2023). 36913864

[17] Farrar, J. T., Polomano, R. C., Berlin, J. A. & Strom, B. L. A comparison of change in the 0-10 numeric rating scaleto a pain relief scale and global medication performance scale in a short-term clinical trial of breakthrough pain intensity. *Anesthesiology.* **112**, 1464–72 10.1097/ALN.0b013e3181de0e6d (2010). PMC2884191 20463579

[18] Feiring, D. C., Ellenbecker, T. S. & Derscheid, G. L. Test-retest reliability of the biodex isokineticdynamometer. *J Orthop Sports Phys Ther* **11**, 298–300 10.2519/jospt.1990.11.7.298 (1990). 18796902

[19] Reinhard, J., Schiegl, J. S., Pagano, S., Leiss, F., Kappenschneider, T., Maderbacher, G., Grifka, J. & Greimel, F. Favourable mid-term isokinetic strength after primary THA combined with a modified enhancedrecovery after surgery concept (ERAS) in a single blinded randomized controlled trial. *Arch Orthop Trauma Surg* **144**, 3323–3336 10.1007/s00402-024-05479-z (2024). PMC11417056 39105846

[20] Wainwright, T. W., Immins, T. & Middleton, R. G. Enhanced recovery after surgery (ERAS) and its applicability for major spinesurgery. *Best Pract Res Clin Anaesthesiol* **30**, 91–102 10.1016/j.bpa.2015.11.001 (2016). 27036606

[21] Zhang, H., Wang, J., Wang, B., Li, K. & Nie, Y. Effect of lower-limb isokinetic muscle strengthening on knee function and joint contact force in knee osteoarthritispatients awaiting total knee arthroplasty: study protocol for a randomized controlled trial. *J Orthop Surg Res* **19**, 677 10.1186/s13018-024-05125-9 (2024). PMC11492781 39434157

[22] Neuprez, A., Delcour, J. P., Fatemi, F., Gillet, P., Crielaard, J. M., Bruyère, O. & Reginster, J. Y. Patients Expectations Impact Their Satisfaction following Total Hip or Knee Arthroplasty. *PLoS One* **11**, e0167911 10.1371/journal.pone.0167911 (2016). PMC5158008 27977711

[23] Khalil, L. S., Darrith, B., Franovic, S., Davis, J. J., Weir, R. M. & Banka, T. R. Patient-Reported Outcomes Measurement Information System (PROMIS) Global Health Short Forms DemonstrateResponsiveness in Patients Undergoing Knee Arthroplasty. *J Arthroplasty* **35**, 1540–1544 10.1016/j.arth.2020.01.032 (2020). 32037211

[24] Roos, E. M., Roos, H. P., Lohmander, L. S., Ekdahl, C. & Beynnon, B. D. Knee Injury and Osteoarthritis Outcome Score (KOOS)--development of a self-administered outcome measure. *J Orthop Sports Phys Ther* **28**, 88–96 10.2519/jospt.1998.28.2.88 (1998). 9699158

[25] Gorišek Miksić, N., Krajnc, Z., Novak, I., Fokter, S. K., Naranđa, J., Moličnik, L. & Moličnik, A. The Development and Implementation of New Recommendations for Perioperative Antibiotic Prophylaxis Duration in Elective Primary Hip and KneeReplacement Surgeries. *J Clin Med* **15**, 3718 10.3390/jcm15103718 (2026). PMC13207017 42194679

[26] Drouin, J. M., Valovich-mcLeod, T. C., Shultz, S. J., Gansneder, B. M. & Perrin, D. H. Reliability andvalidity of the Biodex system 3 pro isokinetic dynamometer velocity, torque and position measurements. *Eur J Appl Physiol* **91**, 22–9 10.1007/s00421-003-0933-0 (2004). 14508689

[27] Wilk, K. E., Arrigo, C. A. & Davies, G. J. Isokinetic Testing: Why it is More Important Today than Ever. *Int J Sports Phys Ther* **19**, 374–80 10.26603/001c.95038 (2024). PMC10987309 38576833

[28] Ju, Y., Wang, G. & Xiao, X. The efficacy ofenhanced recovery program compared to routine care for patients undergoing knee replacement surgery: a systematic review and meta-analysis. *BMCMusculoskelet Disord* **26**, 636 10.1186/s12891-025-08547-6 (2025). PMC12232028 40615790

[29] Wainwright, T. W., Memtsoudis, S. G. & Kehlet, H. Fast-track hip and kneearthroplasty...how fast? *Br J Anaesth* **126**, 348–349 10.1016/j.bja.2020.09.038 (2021). 33478683

[30] Wei, B., Tang, C., Li, X., Lin, R., Han, L., Zheng, S., Xu, Y., Yao, Q. & Wang, L. Enhanced recovery after surgery protocols in total knee arthroplasty via midvastus approach: a randomized controlled trial. *BMCMusculoskelet Disord* **22**, 856 10.1186/s12891-021-04731-6 (2021). PMC8501665 34625057

[31] CheungA, Fu. H., Cheung, M. H., Chan, W. K. V., Chan, P. K., Yan, C. H. & Chiu, K. Y. How well do elderly patients do after total knee arthroplasty in the era of fast-tracksurgery? *Arthroplasty* **2**, 16 10.1186/s42836-020-00037-5 (2020). PMC8796349 35236442

[32] Liao, C., Lai, X., Zhong, J., Zeng, W., Zhang, J., Deng, W., Shu, J., Zhong, H., Cai, L. & Liao, R. Reducing the length of hospital stay for patients undergoing primary total kneearthroplasty by application of enhanced recovery after surgery (ERAS) pathway: a multicenter, prospective, randomized controlled trial. *Eur J Med Res* **30**, 385 10.1186/s40001-025-02647-8 (2025). PMC12079852 40369602

[33] Capin, J. J., Bade, M. J., Jennings, J. M., Snyder-Mackler, L. & Stevens-Lapsley, J. E. Total Knee Arthroplasty Assessments Should Include Strength and Performance-Based Functional Tests to Complement Range-of-Motion and Patient-Reported Outcome Measures. *Phys Ther* **102**, pzac033 10.1093/ptj/pzac033 (2022). PMC9393064 35358318

[34] Bean, J. F., Leveille, S. G., Kiely, D. K., Bandinelli, S., Guralnik, J. M. & Ferrucci, L. A comparison of leg power and leg strength within the InCHIANTI study: which influences mobility more? *J Gerontol A Biol Sci Med Sci.* **58**, 728–33 10.1093/gerona/58.8.m728 (2003). 12902531

[35] Paravlic, A. H., Meulenberg, C. J. & Drole, K. The Time Course of Quadriceps Strength Recovery AfterTotal Knee Arthroplasty Is Influenced by Body Mass Index, Sex, and Age of Patients: Systematic Review and Meta-Analysis. *Front Med (Lausanne)* **9**, 865412 10.3389/fmed.2022.865412 (2022). PMC9174520 35692543

[36] WainwrightTW, GillM., McDonald, D. A., Middleton, R. G., Reed, M., Sahota, O., Yates, P. & Ljungqvist, O. Consensus statement for perioperative care in total hip replacementand total knee replacement surgery: Enhanced Recovery After Surgery (ERAS®) Society recommendations. *Acta Orthop* **91**, 3–19 10.1080/17453674.2019.1683790 (2020). PMC7006728 31663402

